# Potential Effects of an Exoskeleton-Assisted Overground Walking Program for Individuals With Spinal Cord Injury Who Uses a Wheelchair on Imaging and Serum Markers of Bone Strength: Pre-Post Study

**DOI:** 10.2196/53084

**Published:** 2024-01-01

**Authors:** Alec Bass, Suzanne N Morin, Michael Guidea, Jacqueline T A T Lam, Antony D Karelis, Mylène Aubertin-Leheudre, Dany H Gagnon, Montreal Exoskeleton Walking Program (MEWP) Group

**Affiliations:** 1 School of Rehabilitation Faculty of Medicine Université de Montréal Montréal, QC Canada; 2 Centre for Interdisciplinary Research in Rehabilitation of Greater Montreal (CRIR) Centre Intégré Universitaire de Santé et Services Sociaux (CIUSSS) du Centre-Sud-de-l’Île-de-Montréal Montréal, QC Canada; 3 Department of Medicine McGill University Montréal, QC Canada; 4 Department of Exercise Science Faculty of Sciences Université du Québec à Montréal Montréal, QC Canada; 5 Centre de Recherche de l’Institut Universitaire de Gériatrie de Montréal (CRIUGM) Montréal, QC Canada; 6 See Acknowledgements

**Keywords:** assistive technology, bone architecture, bone turnover, osteoporosis, rehabilitation, spinal cord injuries, SCI, spinal cord injury, assistive device, wheelchair, exoskeleton device, locomotion, bone strength, risk, fracture

## Abstract

**Background:**

As many as 60% of individuals use a wheelchair long term after a spinal cord injury (SCI). This mode of locomotion leads to chronic decline in lower-extremity weight-bearing activities and contributes to the development of severe sublesional osteoporosis and high rates of fragility fracture. Overground exoskeleton-assisted walking programs provide a novel opportunity to increase lower-extremity weight bearing, with the potential to improve bone health.

**Objective:**

The aim of the study is to measure the potential effects of an exoskeleton-assisted walking program on lower-extremity bone strength and bone remodeling biomarkers in individuals with chronic (≥18 months) SCI who use a wheelchair.

**Methods:**

In total, 10 participants completed a 16-week exoskeleton-assisted walking program (34 individualized 1-hour sessions, progressing from 1 to 3 per week). Bone mineral density and bone strength markers (dual-energy x-ray absorptiometry: total body, left arm, leg, total hip, and femoral neck and peripheral quantitative computed tomography: 25% of left femur and 66% of left tibia) as well as bone remodeling biomarkers (formation=osteocalcin and resorption=C-telopeptide) were measured before and after intervention and compared using nonparametric tests. Changes were considered significant and meaningful if the following criteria were met: *P*<0.1, effect size ≥0.5, and relative variation >5%.

**Results:**

Significant and meaningful increases were observed at the femur (femoral neck bone mineral content, bone strength index, and stress-strain index) and tibia (cortical cross-sectional area and polar moment of inertia) after the intervention (all *P*<.10). We also noted a decrease in estimated femoral cortical thickness. However, no changes in bone remodeling biomarkers were found.

**Conclusions:**

These initial results suggest promising improvements in bone strength markers after a 16-week exoskeleton-assisted walking program in individuals with chronic SCI. Additional research with larger sample sizes, longer interventions (possibly of greater loading intensity), and combined modalities (eg, pharmacotherapy or functional electrical stimulation) are warranted to strengthen current evidence.

**Trial Registration:**

ClinicalTrials.gov NCT03989752; https://clinicaltrials.gov/ct2/show/NCT03989752

**International Registered Report Identifier (IRRID):**

RR2-10.2196/19251

## Introduction

Mechanical loading is a key factor influencing bone strength [1]. Indeed, osteocytes detect and respond to mechanical stimuli by triggering an anabolic state that stimulates bone formation and leads to adaptations in bone geometry (known as the “mechanostat principle”) [1]. Healthy bones are therefore well adapted to the habitual loads regularly encountered during daily function (ie, concept of specificity) [2]. However, after sustaining a spinal cord injury (SCI), up to 60% of individuals use a wheelchair as their primary mode of locomotion—leading to a chronic reduction in lower-extremity weight bearing and reduced mechanical loading [3]. As a result, these individuals experience an accelerated loss in lower-extremity bone mass, particularly if no mitigation strategies are implemented during the first 18 to 24 months following the SCI [4]. This complication, referred to as sublesional osteoporosis, is associated with an increased risk of fracture, notably at the distal femur and proximal tibia [5].

Bone strength is directly related to fracture risk and can be influenced by several characteristics, such as bone mineral density and content, as well as geometry [6]. Measuring areal bone mineral density by dual-energy x-ray absorptiometry (DEXA) remains widely recommended to assess fracture risk in this population [7]. Indeed, low areal bone mineral density has been associated with increased risks of lower-extremity fractures in individuals with SCI as well as in the general population [8]. However, solely relying on areal bone mineral density to assess bone strength can be misleading since DEXA images display 2D (ie, x- and y-axis) representations of 3D structures (ie, loss of the z-axis) [9]. DEXA condenses structures by superposing images, causing “deeper” bones to artificially appear denser (ie, increased bone mineral density) and may lead to misclassifying individuals with a lower risk of fracture [9]. As such, this limits the DEXA’s capability to inform on bone geometry (eg, cross-sectional areas and cortical thickness) [9,10]. Peripheral quantitative computed tomography (pQCT) aims to overcome this limitation by assessing volumetric bone mineral density based on 3D images [11]. Moreover, pQCT can provide additional advantages by analyzing both trabecular and cortical bone compartments separately (ie, bone geometry) and enable the estimation of mechanical properties of strength (ie, resistivity to compression, bending, and torsion).

Although imaging (DEXA and pQCT) can provide an instantaneous “snapshot” of estimated bone strength, it does not directly assess bone turnover (remodeling). Bone turnover rate can provide fundamental information as to whether bone formation or resorption is dominant at the time of measure. Indeed, serum bone biomarkers (eg, osteocalcin and C-telopeptide) may serve as a precursor indication of a positive therapeutic effect of an intervention, even before changes can be measured with DEXA or pQCT. Osteocalcin is secreted by osteoblasts, is a marker of anabolic bone activity, and has been used in previous studies with individuals with SCI [12]. C-telopeptide, which has also been studied previously in this population, is released during bone resorption and used to characterize catabolic bone activity [13]. Since vitamin D levels can impact bone metabolism, 25-hydroxyvitamin D levels should also be measured as a possible confounding factor when characterizing serum bone biomarkers [7].

Recently, the emergence of wearable robotic exoskeletons has led to new opportunities to develop interventions that can significantly increase lower-extremity weight bearing and mobilization. Among others, a goal of such interventions is to increase bone strength and ultimately mitigate fracture risks (and associated complications) in individuals with SCI. Pilot studies have previously demonstrated that exoskeleton-assisted walking programs are feasible in this population with high rates of satisfaction (95.2%), excellent attendance (ie, 229 completed training sessions out of 234 planned training sessions, 97.9%), and relatively low dropout rates (ie, 1 dropout out of 14 individuals recruited, 7.1%) [14,15]. In terms of learnability and ease of use, most individuals can stand and walk with walking aids and minimal assistance from a therapist by the end of the program (18 to 24 sessions) [15,16]. Walking parameters, including speed and distance, have also been shown to progress consistently and safely over the course of a walking program, especially when individualized progression strategies are used [13,15-19]. Increased walking speed and distance may provide a progressive stimulus for bone strength adaptations, equating to increased intensity and volume for these tissues. Body composition improvements have also been documented following exoskeleton-assisted walking programs, including a decrease in total and regional (ie, lower extremities) body fat and an increase in muscle mass [20]. Overall, these results are encouraging; however, the effects on bone have not been comprehensively evaluated to date.

Thus, the main objective of this paper was to measure the potential effects of a 16-week exoskeleton-assisted walking program on lower-extremity bone density and strength and serum bone turnover markers in individuals with SCI who use a wheelchair [21]. It was hypothesized that immediate positive and meaningful effects would be observed on bone mineral density, mineral content, geometry, and mechanical strength indexes in the lower extremities as well as serum markers of bone turnover (ie, increase in bone formation markers and decrease in bone resorption markers) following the intervention.

## Methods

### Ethical Considerations

Ethics approval for this study was received on March 14, 2019, from the Centre for Interdisciplinary Research in Rehabilitation of Greater Montreal ethics committee (CRIR-1338-0518). The protocol has been published previously and was registered with the US National Library of Medicine on June 7, 2019 (ClinicalTrials.gov NCT03989752) [21].

### Study Design and Participants

This prospective pre- and postinterventional study included adults (≥18 years of age) with chronic (ie, ≥18 months) complete or incomplete SCI. To be included, individuals needed to use a wheelchair as their primary mode of locomotion, understand French or English, and reside (or be able to arrange to reside) within 75 km of the main research site. Individuals were excluded if they had neurological impairments unrelated to the SCI (eg, multiple sclerosis); had a concomitant or secondary musculoskeletal impairment limiting their ability to safely ambulate (eg, hip heterotopic ossification); had a history of fragility fracture within the past year; or had any other condition that may preclude safe lower-extremity weight bearing, walking, or exercise tolerance (eg, unstable cardiovascular or autonomic system and renal insufficiency). Individuals also had to meet criteria specific to the wearable robotic exoskeleton (Ekso GT; Ekso Bionics) used in this study, including maximum anthropometric measures and minimal lower- and upper-extremity range of motion. Inclusion and exclusion criteria are described in greater detail in the published (open access) protocol [21].

### Measurement Times and Intervention

Due to constraints imposed by the COVID-19 pandemic (Multimedia Appendix 1), the 4 measurement times in the published protocol were not possible. Measurement times were only possible before the intervention (2 measurements) and immediately after the intervention (1 measurement). A participant’s preintervention measurements represented the average value between measurements taken before 4 weeks and immediately before initiating the intervention. Postintervention measurements were solely taken immediately following the end of the intervention (ie, within 7 days).

Following preintervention measurements, individuals engaged in a wearable robotic exoskeleton–assisted overground walking program consisting of 34 sessions (60 minutes per session) over a 16-week period. A published algorithm was used to individualize training volume and progression based on osteoporotic profile determined by DEXA [19]. Individuals were classified in 1 of 3 profiles: osteoporosis, osteopenia, or preserved bone mineral density. The number of steps taken per training session was then modulated, starting at 300, 400, and 500, and progressed weekly by 10%, 15%, and 20%, respectively, according to the assigned profile. For all profiles, individuals began with 1 training session per week and progressed to 3 training sessions per week by the end of the program. To maintain a moderate to vigorous exercise intensity during the sessions, walking speed, resting time, assistive devices (ie, walker or crutches), and assistance provided by the therapist were modulated to ensure a rate of perceived exertion of ≥3/10. All training sessions were supervised by a certified physiotherapist, with the help of a second physiotherapist or a physiotherapy technician if necessary.

The exoskeleton-assisted walking program was performed using the Ekso GT exoskeleton. This ready-to-wear exoskeleton has motorized hip and knee joints and semirigid ankle orthoses. Several sensors integrated into the exoskeleton (accelerometers, gyroscopes, pressure sensors, etc) are used to detect weight transfers and movements. Front and lateral spatial targets are used to guide weight transfer with an audible sound emitted when targets are reached. Step initiation depends on the walking mode used. In “FirstStep” mode, front and lateral spatial targets must be reached, followed by the press of a confirmation button by the therapist for stepping movements to be initiated. In “ProStep” mode, stepping is automatically initiated once front and lateral spatial targets are reached (no confirmation button is pressed). In “ProStep+” mode, the lateral spatial target must be reached (no front target is necessary), and the participant must initiate a hip flexion moment to activate stepping. Additionally, the exoskeleton also provides different levels of assistance, from partial (the participant must generate some lower extremity force, and the exoskeleton assists as required) to maximal (the participant does not generate lower extremity force, and the exoskeleton realizes all movements).

### Outcomes

#### DEXA Measurement

Total body, lumbar, and left hip mineral density and content were measured using DEXA (General Electric Lunar Prodigy; standard mode; version 12.30.008). Calibration was executed daily with a standard phantom prior to each test. Participants were asked to fast for at least 8 hours prior to the assessment. Participants were also asked to empty their bladder if they had not done so within the hour preceding the DEXA. Scans were taken following the standardized protocol recommended by the manufacturer. For all scans, participants lay supine, free of jewelry or any other metallic objects. Clothing worn was noted, and participants were asked to wear the same clothing for repeated scans. For lumbar scans, participants’ lower extremities rested on a block to maintain a flexed-hip position and reduce lumbar lordosis, as recommended by the Centers for Disease Control and Prevention [22]. For hip scans, a triangular bracing device attached to the feet maintained the lower extremity in slight internal rotation, as recommended by the Centers for Disease Control and Prevention [22]. Quantitative analysis was provided automatically by the manufacturer’s software. Total body, L4 lumbar vertebrae, left arm, left leg, left total hip, and left femoral neck bone mineral densities and contents were selected as outcomes of interest. Total body measurements provided an estimate of the whole skeletal system. Lumbar vertebrae and left arm measurements provided comparators for lower extremity measurements, as changes were not expected to occur at these sites. Left leg measurements provided an estimate of the overall response of the lower extremities, which complemented the more specific pQCT measurements (described hereafter). Total hip and femoral neck sites provided a comparator with the broader osteoporosis literature, as these remain standard measurements for all populations with osteoporosis. When applicable, the left side of the body was selected to match with the pQCT scan sites.

#### pQCT Measurement

All pQCT imaging was realized on the left distal femur and proximal tibia. A standardized scan protocol was developed based on previous recommendations [11]. Calibration was executed daily with a standard phantom prior to each test. For all scans, a voxel size of 0.5×0.5 mm was used, and the scan speed was set to 10 mm/s to optimize resolution for bone and soft tissues. The total length was measured manually for the femur from the lateral femoral condyle to the greater trochanter [11]. To ensure location consistency for repeated scans, scout scans were realized at the knee joint with a reference line placed at the distal limit of the lateral femoral condyle. Following the scout scan, the pQCT was programmed to take one 2-mm slice at 25% of the total bone length calculated from the reference line. For the tibia, the total length was measured manually from the medial malleolus to the medial plateau [11]. To ensure location consistency for repeated scans, scout scans were realized at the knee joint with a reference line placed at the most distal and flattest portion of the tibial plateau. Following the scout scan, the pQCT was programmed to take one 2-mm slice at 66% of the total length calculated from the distal limit of the bone (using the reference line in this study, this equates to 33% from the knee joint). Both sites were selected to optimize for the presence of both bone and soft tissues in the scans.

Prior to quantitative analysis, the quality of all pQCT images was independently assessed by 2 evaluators (AB and MG or JTATL) using a previously published 5-level visual inspection and quality scale, where an image score of 1 indicated high quality and an image score of 5 represented low quality [23]. To further standardize the assessment of image quality, the following criteria were agreed upon between evaluators: score 1, if the image was free of movement artifacts; score 2, if the image was only a few movement artifacts; score 3, if the image had several movement artifacts, but periosteum continuity was not affected; score 4, if the image had several movement artifacts, and periosteum continuity was affected; and score 5, if the image had movement artifacts leading to complete loss of bone continuity. A mean score was calculated for each image. Scans with a mean score greater than 3 were excluded, as such quality of the image has been proposed to be incompatible with quantitative analysis software [23]. Excluded images were treated as missing data, and measurements were computed following an intention-to-treat protocol.

Quantitative analysis of pQCT scans was realized using the manufacturer’s software (Stratec XCT-3000; version 6.20). For all scans, contour mode 3 with a threshold set to 130 mg/cm^3^, peel mode 2 set to 400 mg/cm^3^, and separation mode 4 with an outer threshold of 200 mg/cm^3^ and an inner threshold of 650 mg/cm^3^ were used [11]. Outcomes of interest were those related to bone mineral density (total, trabecular, and cortical), bone mineral content (total, trabecular, and cortical), bone geometry (cross-sectional areas and cortical thickness), and mechanical strength indexes (bone strength index, stress-strain index, and polar moment of inertia) [7,11].

The software provides 2 measurements for cortical thickness. The first (CRT_THK), referred hereafter as measured cortical thickness, is the mean cortical thickness based on an iterative algorithm that attempts to draw the endosteal and periosteal borders by consecutively comparing neighboring voxels (pixels). Due to occasional failure of the algorithm, particularly in individuals with severe cortical thinning and loss of cortical bone mineral density (ie, many individuals with chronic SCI), the software also provides a second measurement. This measurement (CRT_THK_C), referred hereafter as estimated cortical thickness, is based on a subtraction of endosteal radius from periosteal radius in a theoretical circular model, where total and trabecular cross-sectional areas match those measured. Since measured cortical thickness systematically failed in 2 participants, estimated cortical thickness is also reported in this study.

**Figure 1:**
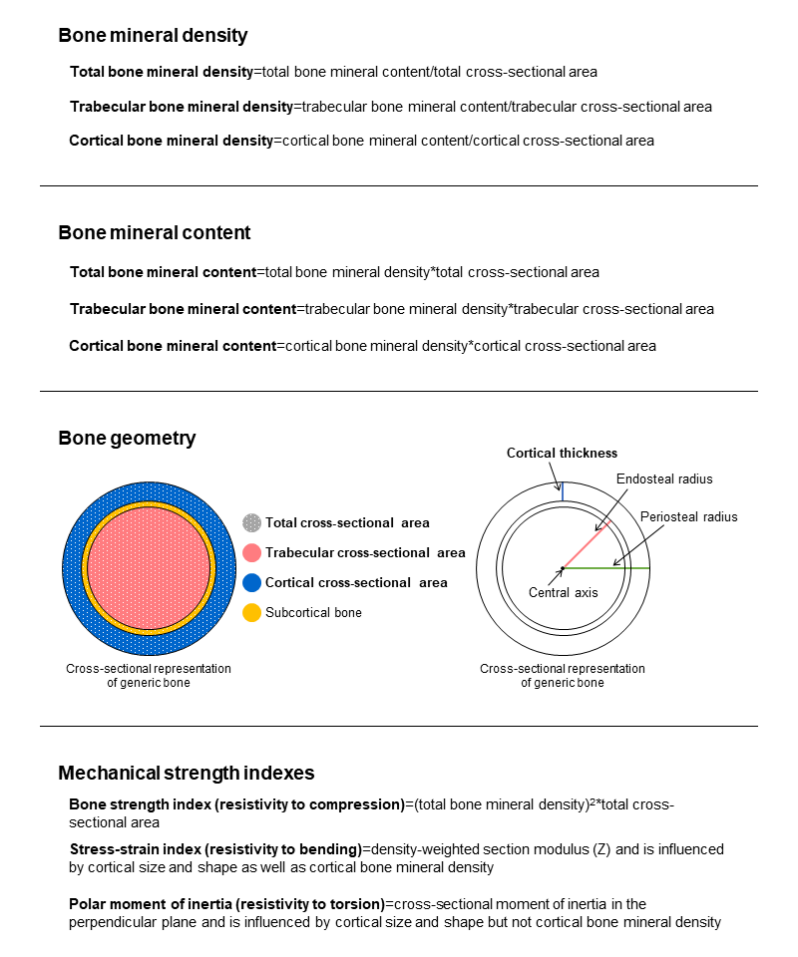
Summary of, and relationships between, outcomes of interest for peripheral quantitative computed tomography.

Estimations of mechanical strength indexes are based on material properties and are calculated as follows. The bone strength index is the product of total bone mineral density squared by total cross-sectional area (ie, bone strength index = total bone mineral density^2^ × total cross-sectional area) and is indicative of resistance to compression [10,24]. The stress-strain index (resistivity to bending) is based on the calculation of the cross-sectional moment of inertia (ie, area moment of inertia or second moment of area) [10,24]. The cross-sectional moment of inertia considers the distance of cortical bone from the central axis of the bone. The greater the distance separating cortical bone from the central axis, the greater the resistivity. To calculate the stress-strain index, section modulus (Z) is computed from the cross-sectional moment of inertia in the transversal plane. Section modulus is then weighted against measured cortical bone mineral density. Thus, resistance to bending is influenced by cortical size, shape, and mineral density [10,24]. Polar moment of inertia is based on the calculation of the cross-sectional moment of inertia in the longitudinal plane [10,24]. Thus, resistance to torsion is influenced by cortical size and shape but not mineral density [10,24]. The pQCT-related variables of interest and their cross-relationships are summarized in Figure 1.

#### Blood Samples

Blood samples were drawn in the morning, following an 8-hour fast, by a licensed nurse into gold-top serum separator and lavender-top anticoagulant ethylenediaminetetraacetic acid tubes. Samples were immediately placed on ice and centrifuged within an hour. Serum (from gold-top serum separator tubes) and plasma (from lavender-top anticoagulant ethylenediaminetetraacetic acid tubes) were collected and stored at –80 °C until analysis. Blood samples were transported on dry ice to a university hospital laboratory at the McGill University Health Centre for analysis after the completion of the study. Serum was used to measure 25-hydroxyvitamin D, and plasma was used to measure osteocalcin and C-telopeptide.

### Statistics

Descriptive statistics were used to characterize participants. Since the sample size was limited and some outcome measures were not normally distributed, nonparametric tests (ie, Wilcoxon signed rank test) were used to compare pre- versus postintervention data. Standardized effect sizes (*r*) were calculated by dividing the *z* value by the square root of the number of observations and interpreted as being negligible (<0.1), small (≥0.1), medium (≥0.3), or large (≥0.5) [25]. Relative pre- versus postintervention median variations (%) were also computed for all outcomes. Given the explorative nature of this study, three criteria needed to be met to reach significance and meaningfulness: (1) the α for statistical tests needed to be <.10 to balance the risk of false negatives due to an anticipated lack of statistical power, (2) calculated effect sizes needed to be large (ie, ≥0.5) for an outcome to be deemed potentially clinically relevant, and (3) relative variation needed to be greater than 5% to be considered as a change exceeding natural variability and potential measurement errors. This threshold has been used in previous work, as the least significant change reportedly varies between 2% and 5% for DEXA and pQCT depending on the location of the scan [12,26]. All statistical analyses were conducted using SPSS (version 28; IBM Corp).

## Results

### Overview

Characteristics of the participants are summarized in Table 1. Among the 10 participants, only 1 had a very minimal motor function in the lower extremities (lower-extremity motor score: 5 out of 50), although it was not sufficient for active participation of the lower extremities during the exoskeleton-assisted walking program. Therefore, the exoskeleton was programmed to detect body weight shifts and realize stepping movements without active participation of the lower extremities (“ProStep” mode with maximal assistance in the exoskeleton) for all participants.

**Table 1:** Description of the participants (N=10).

Participant ID	Sex	Age (y)	BMD profile^a^	Walking program progression	Neurological lesion level	AIS^b^	LEMS^c^	Exoskeleton mode (Ekso GT)	SCI^d^ duration (y)	Weight (kg)	Height (m)	BMI (kg/m^2^)	Total body fat (%)^e^
1	Male	41	Preserved	Fast	T8	A	0	ProStep	9.6	66.7	1.71	22.8^f^	34.1^f^
2	Male	36	Preserved	Fast	T6	A	0	ProStep	11.6	99.7	1.92	27.0^f^	39.5^f^
3	Male	67	Preserved	Fast	T10	A	0	ProStep	12.0	92.3	1.88	26.1^f^	37.8^f^
4	Male	60	Preserved	Fast	T11	A	0	ProStep	3.3	90.6	1.74	29.9^f^	38.7^f^
5	Female	35	Preserved	Fast	C3	C	0	ProStep	3.6	50.2	1.65	18.4	29
6	Male	32	Osteopenia	Moderate	T3	A	0	ProStep	8.6	73.5	1.75	24.0^f^	24.6^f^
7	Female	48	Osteopenia	Moderate	T12	B	5	ProStep	45.5	62.4	1.60	24.4^f^	51.8^f^
8	Female	42	Osteopenia	Moderate	T3	A	0	ProStep	7.7	70.7	1.66	25.7^f^	44.4^f^
9	Female	55	Osteoporosis	Slow	T4	A	0	ProStep	7.8	61.2	1.66	22.2^f^	43^f^
10	Male	47	Osteoporosis	Slow	C5	A	0	ProStep	18.3	81.3	1.86	23.5^f^	42.7^f^
Mean (SD)	N/A^g^	46.3 (10.9)	N/A	N/A	N/A	N/A	N/A	N/A	12.8 (11.6)	74.9 (15.0)	1.70 (0.10)	24.4 (2.9)	38.5 (7.4)

^a^BMD profile: preintervention bone mineral density profile of the left hip as measured by dual-energy x-ray absorptiometry (DEXA).

^b^AIS: American Spinal Injury Association Impairment Scale.

^c^LEMS: lower-extremity motor score on the AIS.

^d^SCI: spinal cord injury.

^e^Total body fat percentage as measured by DEXA.

^f^Identifies obesity using criteria recommended by Paralyzed Veterans of America (BMI≥22 kg/m^2^ or body fat>22% in men and >35% in women) [27].

^g^N/A: not applicable.

### DEXA Outcome Measures

Outcome measures for DEXA are summarized in Table 2. Only the left femoral neck bone mineral content met all 3 criteria with a *P*=.08, a large effect size (0.55), and a relative increase of 6% postintervention.

**Table 2:** Summary of dual-energy x-ray absorptiometry outcome measures (N=10).

Outcomes			Preintervention, median (IQR)		Postintervention, median (IQR)		*P* value	Effect size^a^		∆^b^ (%)
**Areal bone mineral densities (g/cm^2^)**										
	Total body bone mineral density	1.159 (1.060-1.277)		1.145 (1.082-1.267)		.80		0.08 (N)	–1.2	
	Left arm bone mineral density	1.046 (0.909-1.155)		1.073 (0.889-1.221)		.51		0.20 (S)	+2.6	
	Left leg bone mineral density	1.018 (0.613-0.898)		0.979 (0.442-0.902)		.45		0.24 (S)	–3.8	
	Left total hip bone mineral density	0.862 (0.756-0.992)		0.832 (0.755-0.989)		.68		0.13 (S)	–3.4	
	Left femoral neck bone mineral density	0.852 (0.765-0.992)		0.908 (0.770-0.947)		.11		0.50 (L)	+6.6	
**Bone mineral contents (g/cm)**										
	Total body bone mineral content	2759 (2377-3499)		2757 (2365-3466)		.33		0.31 (M)	–0.1	
	Left arm bone mineral content	188 (174-236)		202 (173-241)		.65		0.15 (S)	+7.3	
	Left leg bone mineral content	393 (300-510)		370 (312-528)		.80		0.08 (N)	–5.9	
	Left total hip bone mineral content	28.3 (20.8-34.9)		32.1 (20.2-36.7)		.39		0.27 (S)	+13.5	
	*Left femoral neck bone mineral content^c^*	4.5 (3.5-6.0)		4.8 (3.6-5.9)		*.08^d^*		*0.55 (L)*	*+6*	

^a^Standardized effect sizes interpreted as N=negligible (<0.1), S=small (≥0.1), M=medium (≥0.3), or L=large (≥0.5).

^b^∆=relative variation between medians (positive indicates an increase in value from pre- to postmeasurement).

^c^Italics format indicates variables meeting the following 3 criteria: statistically significant difference, effect size ≥0.5, and relative median difference ≥5%.

^d^Statistically significant difference (*P*≤.10) for Wilcoxon signed rank tests.

### pQCT Outcome Measures

For the femur, outcome measures for pQCT are summarized in Table 3. Although 9 outcomes were statistically significant (*P*<.10), only 3 had large effect sizes and sufficient relative changes to be considered as intervention effects. Bone strength index (resistivity to compression; *P*=.09) and stress-strain index (resistivity to bending; *P*=.01) increased by 9.6% and 11%, respectively, whereas estimated cortical thickness (*P*=.01) decreased by 9.9%. Of note, scans at the femur were not possible for 1 participant (participant 10), as his weight and lack of core stability impeded his ability to safely take and maintain the crouched sitting position necessary to set up the femur into the pQCT.

For the tibia, outcome measures for pQCT are summarized in Table 4. Although 6 outcomes were statistically significant (*P*<.10), only 2 had large effect sizes and sufficient relative changes to be considered potential intervention effects. Cortical cross-sectional area (*P*=.06) and polar moment of inertia (*P*=.01) increased by 7.3% and 5.1%, respectively.

**Table 3:** Summary of peripheral quantitative computed tomography outcome measures at 25% of the left femur (n=9).

Outcomes			Preintervention, median (IQR)		Postintervention, median (IQR)		*P* value		Effect size^a^		∆^b^ (%)
**Volumetric bone mineral densities (mg/cm^3^=)**											
	Total bone mineral density	355.8 (334.2-470.5)		381.6 (330.8-442.6)		.51		0.22 (S)		+7.3	
	Trabecular bone mineral density	87.7 (80.5-113.0)		88.5 (83.6-110.0)		.15		0.22 (S)		+1	
	Cortical bone mineral density	905.9 (805.0-968.1)		938.2 (871.5-981.6)		.04^c^		0.69 (L)		+3.6	
**Bone mineral contents (mg/mm)**											
	Total bone mineral content	346 (275-434)		341 (266-429)		.05^c^		0.65 (L)		–1.5	
	Trabecular bone mineral content	46.6 (37.9-76.7)		48.0 (39.1-78.4)		.95		0.02 (N)		+3	
	Cortical bone mineral content	275 (224-350)		268 (217-343)		.01^c^		0.89 (L)		–2.5	
**Bone geometry**											
	Total cross-sectional area (mm^2^)	822 (736-1066)		805 (770-1023)		.14		0.49 (L)		–2	
	Trabecular cross-sectional area (mm^2^)	489 (418-700)		472 (435-659)		.46		0.25 (S)		–3.4	
	Cortical cross-sectional area (mm^2^)	312 (233-394)		305 (221-354)		.01^c^		0.89 (L)		–2.4	
	Measured cortical thickness (n=7; mm)	4.03 (3.56-4.28)		3.88 (3.31-4.23)		.03^c^		0.83 (L)		–3.6	
	*Estimated cortical thickness (mm)* ^d^	3.28 (2.89-3.44)		2.95 (2.95-3.35)		*.01* ^c^		*0.85 (L)*		–*9.9*	
**Mechanical strength indexes**											
	*Compression: bone strength index (g/cm^4^)*	1.35 (1.16-1.60)		1.48 (0.94-1.51)		*.09* ^c^		*0.57 (L)*		*+9.6*	
	*Bending: stress-strain index (mm^3^)*	2240 (2047-2589)		2486 (2356-2706)		*.01* ^c^		*0.89 (L)*		*+11*	
	Torsion: polar moment of inertia (mm^4^)	48,002 (43,337-72,759)		48,800 (42,470-71,304)		.02^c^		0.77 (L)		+1.7	

^a^Standardized effect sizes interpreted as N=negligible (<0.1), S=small (≥0.1), M=medium (≥0.3), or L=large (≥0.5).

^b^∆=relative variation between medians (positive indicates an increase in value from pre- to postmeasurement).

^c^Statistically significant difference (*P*≤.10) for Wilcoxon signed rank tests.

^d^Italics format indicates variables meeting the following 3 criteria: statistically significant difference, effect size ≥0.5, and relative median difference ≥5%.

**Table 4:** Summary of peripheral quantitative computed tomography outcome measures at 66% of the left tibia (N=10).

Outcomes			Preintervention, median (IQR)		Postintervention, median (IQR)		*P* value		Effect size^a^		∆^b^ (%)
**Volumetric bone mineral densities (mg/cm^3^)**											
	Total bone mineral density	666.0 (571.1-772.6)		669.2 (554.0-772.4)		.06^c^		0.60 (L)		+0.5	
	Trabecular bone mineral density	97.3 (86.0-105.9)		95.0 (81.3-109.5)		.14		0.47 (M)		–2.4	
	Cortical bone mineral density	984.9 (961.0-1007.9)		956.4 (898.2-1004.8)		.07^c^		0.56 (L)		–2.9	
**Bone mineral contents (mg/mm)**											
	Total bone mineral content	326 (288-425)		333 (292-427)		.14		0.47 (M)		+2.3	
	Trabecular bone mineral content	20.1 (12.5-24.5)		18.0 (13.1-24.4)		.88		0.05 (N)		–10.1	
	Cortical bone mineral content	283 (264-394)		288 (270-398)		.09^c^		0.53 (L)		+1.9	
**Bone geometry**											
	Total cross-sectional area (mm^2^)	602 (425-621)		610 (423-660)		.06^c^		0.60 (L)		+1.4	
	Trabecular cross-sectional area (mm^2^)	224 (124-274)		217 (124-295)		.34		0.50 (L)		–3	
	*Cortical cross-sectional area (mm^2^)* ^d^	294 (267-388)		315 (273-420)		*.06* ^c^		*0.60 (L)*		*+7.3*	
	Measured cortical thickness (n=8; mm)	5.22 (4.74-5.67)		5.31 (4.86-5.53)		.12		0.54 (L)		+1.8	
	Estimated cortical thickness (mm)	4.80 (3.96-5.48)		4.70 (4.26-5.78)		.33		0.31 (M)		–2.1	
**Mechanical strength indexes**											
	Compression: bone strength index (g/cm^4^)	2.06 (1.67-2.85)		2.03 (1.63-2.88)		.20		0.40 (M)		–1.5	
	Bending: stress-strain index (mm^3^)	1838 (1346-2294)		1828 (1300-2250)		.58		0.18 (S)		–0.5	
	*Torsion: polar moment of inertia (mm^4^)*	35,706 (23,560-47,987)		37,539 (23,638-49,806)		*.01* ^c^		*0.79 (L)*		*+5.1*	

^a^Standardized effect sizes interpreted as N=negligible (<0.1), S=small (≥0.1), M=medium (≥0.3), or L=large (≥0.5).

^b^∆=relative variation between medians (positive indicates an increase in value from pre- to postmeasurement).

^c^Statistically significant difference (*P*≤.10) for Wilcoxon signed rank tests.

^d^Italics format indicates variables meeting the following 3 criteria: statistically significant difference, effect size ≥0.5, and relative median difference ≥5%.

### Serum Bone Turnover Biomarkers

Outcome measures for serum bone turnover biomarkers are summarized in Table 5. Only 25-hydroxyvitamin D met all 3 criteria with a *P*=.03, a large effect size, and a relative increase of 11.4% postintervention.

**Table 5:** Summary of serum bone turnover biomarkers (N=10).

Outcomes			Preintervention, median (IQR)	Postintervention, median (IQR)		*P* value		Effect size^a^		∆^b^ (%)
**Bone formation (μg/L)**										
	Osteocalcin	18.3 (15.6-19.4)		21.0 (15.3-24.0)	.20		0.69 (L)		+15.1	
**Bone resorption (μg/L)**										
	C-telopeptide	0.3 (0.2-0.4)		0.3 (0.2-0.4)	.17		0.43 (M)		–13.8	
**Others (nmol/L)**										
	*25-Hydroxyvitamin D^c^*	74.5 (62.4-111)		83.0 (66.3-129)	*.03* ^d^		*0.69 (L)*		*+11.4*	

^a^Standardized effect sizes interpreted as N=negligible (<0.1), S=small (≥0.1), M=medium (≥0.3), or L=large (≥0.5).

^b^∆=relative variation between medians (positive indicates an increase in value from pre- to postmeasurement).

^c^Italics format indicates variables meeting the following 3 criteria: statistically significant difference, effect size ≥0.5, and relative median difference ≥5%.

^d^Statistically significant difference (*P*≤.10) for Wilcoxon signed rank tests.

## Discussion

### Principal Findings

Results of this preliminary study indicate that the completion of a progressive 16-week exoskeleton-assisted walking program may elicit some beneficial bone adaptations in individuals with chronic SCI who have limited-to-no motor function in their lower extremities and use a manual wheelchair as their primary mode of locomotion.

### DEXA Revealed an Increase in Left Femoral Neck Bone Mineral Content, but No Changes in Bone Mineral Densities

Left femoral neck bone mineral content increased significantly and meaningfully following the intervention which is, to our knowledge, a novel and key finding partly supporting our hypotheses. Moreover, a similar trend (ie, *P*=.11) was also observed in left femoral neck bone mineral density (ie, +6.6% with a large effect size). Indeed, since bone mineral content and density are directly related (ie, bone mineral density = bone mineral content / area), it would be expected for both to change together. Directly comparing our results to the literature remains difficult due to the lack of previously published evidence. This is particularly true with regard to bone mineral content, as this outcome has not been reported in the limited available literature with regard to exoskeleton-assisted overground walking and treadmill-based interventions [12,20,28-32].

Nevertheless, with regard to exoskeleton-assisted overground walking, a pilot study conducted in our laboratory did not reveal any significant changes in total body and total leg areal bone mineral densities, which is consistent with this study [20]. To our knowledge, only 2 other studies have reported areal bone mineral density measurements following exoskeleton-assisted overground walking. First, in a pilot study, an upward trend in areal bone mineral density was reported following 8 weeks of training (1 hour per session, 2 sessions per week). However, the authors neither specify in what body region this occurred nor present data to support this claim [28]. Second, in a pilot randomized controlled trial, including 16 participants with SCI (≥2 years) who use a wheelchair, areal bone mineral density (total hip and femoral neck) decreased in the activity-based exercise training group (60 minutes per session, 3 sessions per week for 24 weeks), whereas it remained stable in the exoskeleton-assisted walking group (60 minutes per session, 3 sessions per week for 24 weeks). It was hypothesized that exoskeleton-assisted walking may provide a sufficient stimulus to maintain areal bone mineral density but perhaps not to augment it [29]. Since this study did not include a comparison group, it remains unclear whether the areal bone mineral densities measured in our participants would have decreased further over the course of the study had they not participated in the walking program. However, all participants in this study sustained their SCI at least 3 years before initiating the study and were deemed to have reached a stable state in terms of bone mineral density. To this effect, it is now well evidenced that bone loss is greatest within the first 18 to 24 months following the lesion and tends to slow considerably thereafter [4]. Although a true steady state in bone mass may never be reached, it would be premature to state that the intervention in this study had a protective effect on areal bone mineral density [33]. Such a hypothesis would be best tested by recruiting participants who recently sustained their SCI (ie, no more than 2 years prior) and including a comparison group.

The effects of treadmill-based walking programs have also been reported in the literature using robotic assistance (eg, Lokomat; Hocoma), functional electrical stimulation, or manual assistance [12,30,31]. To our knowledge, no study has reported bone mineral content, and no changes in areal bone mineral density have been previously found [12,30-32]. Since these programs imply the use of partial body weight support, the gravity-related mechanical effects decreased considerably in comparison to overground walking, which may impede the effectiveness of such programs. This is further highlighted by the fact that treadmill-based walking programs have also been tested in combination with pharmacotherapy (ie, teriparatide) and functional electrical stimulation, which should have optimized the potential effects on bone [12,30].

Overall, this study suggests that exoskeleton-assisted overground walking may elicit a beneficial bone response at the hip that can be detected by DEXA. A combination of pharmacotherapy (eg, teriparatide), functional electrical stimulation, and overground walking may be needed to provide an optimal anabolic stimulus to significantly increase areal bone mineral density, and this warrants consideration for future research.

### Potential Improvements in Bone Strength as Measured by pQCT

A few pQCT outcomes changed significantly and meaningfully following the completion of the intervention. This result supports our hypotheses in part. Four such outcomes increased, suggesting positive bone strength adaptations: femoral bone strength index (compression), femoral stress-strain index (bending), tibial cortical cross-sectional area, and tibial polar moment of inertia (torsion).

With regard to the femur, to our knowledge, the increase in bone strength index is a novel finding [12,20,30-32,34]. However, an increase in stress-strain index has been previously reported in a case study following robotic-assisted treadmill training [34]. Yet, the amplitude of change reported in this previous case study (right femur=+2% and left femur=+0.5%) was much lower than in this study (ie, +11%), and may not have exceeded natural variability or measurement error. Nevertheless, these findings highlight the importance of including both femoral and tibial measurements with pQCT in this population. Since bone is expected to respond in areas of greatest mechanical strain, certain biomechanical concepts may help partially explain the results in this study [33]. First, although the increase in bone strength index would be expected with increased weight-bearing, the design of the exoskeleton may also contribute to greater compression forces at the femur during heel strike. Indeed, the exoskeleton used in this study uses a brace at the proximal tibia, just below the knee, to counteract the forward velocity of the lower limb (and body) during heel strike. Since the individuals in this study had very little-to-no motoricity in the lower limbs, this forward velocity could not be absorbed to the same extent by musculotendinous structures (ie, through eccentric contraction of the quadriceps) and would therefore be mainly absorbed by the skeletal (ie, femur) and ligamentous structures [35]. Second, due to the oblique orientation of the femoral diaphysis, it is possible that the forces with heel strike and unilateral stance during walking provide greater strain (ie, bending force) to the femur than the tibia, which may have also contributed to the results in this study [36]. Overall, these hypotheses warrant further investigation.

With regard to the tibia, changes in cortical cross-sectional area and polar moment of inertia have been previously reported in 2 treadmill-based interventions [12,34]. However, the relatively small amplitudes of changes in these previous studies (ie, –1 to +1.4%) raise questions as to whether these changes can be attributed to more than natural measurement error. In fact, in one of these studies, comparisons with a control group yielded no significant difference for polar moment of inertia (cortical cross-sectional area was not reported in this study) [12]. Interestingly, we have previously hypothesized that the design of current exoskeletons may limit the automatic external rotation of the tibia on the femur (and consequently, the foot) during knee extension [37]. This may have led to increased torsion moments in the tibia, which would not occur during treadmill walking without robotic assistance (ie, knee extension in an open kinetic chain)—and could partially explain the difference in amplitude of change between studies.

### Uncertainties Remain Regarding pQCT Outcomes

The fact that the estimated femoral cortical thickness decreased (–9.9%) in this study, which does not align with our hypotheses, could raise concerns regarding the possible negative effects of the walking program on bone strength. Indeed, cortical bone is largely believed to be the primary source of resistance and strength for long bones, such as the femur and tibia [9,10]. To our knowledge, these results have not been previously reported in the femur. In 1 treadmill-based trial, a statistically significant reduction of cortical thickness was reported in the tibia [12]. However, this reduction only occurred 8 months following the completion of the training program and was not statistically different than that of the control group [12]. Of interest, a statistically significant reduction in cortical cross-sectional was also observed in this study, which most likely is explained by natural variability or measurement error, considering the relatively small magnitude of change (–2.4%). Moreover, when compared to men without SCI, individuals with SCI show reductions in cortical cross-sectional area of approximately 34% [38]. Thus, the clinical significance of a 2.4% reduction in this parameter remains questionable. Nevertheless, reductions in cortical thickness and cross-sectional area may suggest that the analysis software assigned a larger proportion of bone as subcortical (identified in yellow in Figure 1), which could be related to changes in density (ie, increased porosity) at the endosteal border due to bone resorption. This possibility cannot be completely excluded from the results of this study, particularly when considering the small sample size and the limited statistical power. Future studies should pay special attention to the possible negative effects on cortical thickness and cross-sectional area at the femur.

### Serum Biomarkers Were Not Able to Contextualize pQCT Findings, but an Unexpected Increase in Levels of Serum Vitamin D Occurred

Serum osteocalcin (bone formation) and C-telopeptide (bone resorption) did not change significantly between before and after the intervention. This provides further evidence with regard to the complexity of the interpretation of the pQCT findings, as it is not immediately obvious whether increased bone formation or resorption was occurring following the intervention. These results were not anticipated, as 4 months of treadmill walking combined with functional electrical stimulation has been shown to significantly increase osteocalcin (+6.4%) and reduce C-telopeptide (–7.7%) levels in individuals with chronic SCI [12]. The variations found in this study (ie, osteocalcin=+15.1% and C-telopeptide=–13.8%) present trends of similar direction and of greater amplitude when compared to those previously reported, although the statistical threshold was not reached.

Serum vitamin D (25-hydroxyvitamin D) increased significantly and meaningfully by 11.4% during the intervention. Although higher vitamin D levels have been associated with greater levels of physical activity, this is generally attributed to increased time exposed to the sun in more active individuals [39]. In this study, all participants were educated regarding vitamin D supplementation recommendations by Osteoporosis Canada [40]. Participants who were not already taking vitamin D (4/10) were offered 1 year’s worth of oral supplementation. Only 1 participant began taking vitamin D supplementation during the 4-week period before initiating training. However, even when removing this participant, the data remained statistically significant (*P*=.05). A possible explanation for this finding is the fact that most training sessions were delivered during the transition from winter to summer months. It is well recognized that vitamin D levels tend to be lower during winter months in northern countries such as Canada, as individuals spend more time indoors [41]. Thus, it is possible that the timing of the study coincided with an expected increase in vitamin D levels seen in the general population during the transition from winter to summer [41]. Nevertheless, serum 25-hydroxyvitamin D levels remained within optimal ranges (ie, ≥75 nmol/L) throughout the duration of the study [42]. As such, bone turnover and metabolism are not expected to have been significantly affected. Moreover, vitamin D supplementation, on its own, has not been shown to effectively increase bone mineral density [43]. Therefore, it is not expected that the variations in bone markers in this study can be attributed to the measured changes in serum 25-hydroxyvitamin D levels.

### Limitations and Future Perspectives

This study has limitations that warrant consideration when interpreting its results. First, the sample size was smaller than that initially planned due to numerous challenges associated with the COVID-19 pandemic. Consequently, this reduced statistical power and increased the chance of potential type 2 errors (ie, false negatives). Moreover, the relatively small sample size impeded the possibility of conducting additional subgroup analysis. For example, it was not possible to compare participants according to clinical characteristics (eg, gender, osteoporotic status, obesity status, and response to intervention). Unfortunately, this limits progress toward a more personalized approach for the proposed intervention. Second, the absence of bone mineral density–based inclusion or exclusion criteria led to the recruitment of 5 participants (50% of the sample size) with “preserved” bone mineral density. Hence, these participants were inherently less inclined to benefit from the walking program in terms of bone health. Third, this study did not have specific inclusion or exclusion criteria for concomitant bone health treatments. However, a complete list of medications was taken for each participant, and they were instructed to inform the research team if any changes in medications occurred during the project. Of note, none of the participants were receiving antiosteoporosis agents at the time of the study. Participants were also asked to maintain their physical activity levels during the duration of the study, including their regular exercise regime. Fourth, this study did not have a control group, as such, results should be interpreted with caution as it is unknown to what extent the absence of (or relatively small) changes measured would differ from natural variability in time. Finally, the intensity and duration of the intervention may have been insufficient. Bone resorption typically lasts 30 to 40 days, whereas bone formation frequently requires an additional 150 days, for a total bone turnover cycle requiring up to 6 months [10]. Therefore, it is plausible that clinically significant changes in bone strength could take up to 6 months, indicating that the 4-month measurement period in this study may not have been sufficient. For instance, interventions of 6 or more months, with stationary cycling assisted by functional electrical stimulation, have measured positive effects on bone mass, whereas shorter interventions have not [44-50]. Moreover, despite being initially planned, no follow-up assessments were authorized due to the COVID-19 pandemic, and the beneficial changes that may have emerged later in relation to the temporality of bone adaptation were not captured.

Future research should focus on larger sample sizes, with a particular interest on individuals most likely to benefit from the intervention (ie, individuals with reduced bone mass). From a pragmatic perspective, large multicentric trials will be most likely required to have a sufficient sample size to detect a 5% change in femoral bone mineral density (pQCT) and compensate for large natural heterogeneity in this population. In fact, using the data in this study, this most likely entails the recruitment of roughly 200 participants based on Lehr equation (n=8*s*^2^/*δ*^2^). Interventions should be of sufficient volume (ie, at least 3 times per week), possibly of greater intensity, and of medium- to long-term durations (ie, at least 6 months) to ensure adequate stimulus and time for complete bone turnover cycles. Follow-up assessments, after the completion of the intervention, are also warranted to assess possible latent adaptations. The addition of a control group also remains relevant to compensate for natural variability and measurement error related to bone imaging and serum sampling. Finally, combining pharmacological interventions (eg, teriparatide) or functional electrical stimulation or both with overground exoskeleton–assisted walking may also warrant consideration.

### Conclusions

The results from this paper confirm that a 16-week exoskeleton-assisted walking program may elicit bone adaptations. On one hand, significant and meaningful increases were documented via DEXA and pQCT at both the femur (ie, femoral neck bone mineral content, bone strength index, and stress-strain index) and tibia (ie, cortical cross-sectional area and polar moment of inertia). On the other hand, possible significant and meaningful decreases (ie, femoral cortical thickness) raise concerns. Although positive bone adaptations are emerging, it remains unclear whether completing a 16-week exoskeleton-assisted walking program increases bone strength in individuals with chronic SCI. The need for stronger evidence warrants additional research with larger sample sizes that focus on longer interventions (possibly of greater loading intensity), and combining modalities should be considered (eg, pharmacotherapy or functional electrical stimulation). To do so, national or international collaborations will most likely be required.

## Appendix

Multimedia Appendix 1 Project timeline and effects of the COVID-19 pandemic.

## Abbreviations

DEXA: dual-energy x-ray absorptiometry
pQCT: peripheral quantitative computed tomography
SCI: spinal cord injury
