# Supplementary figures and images for: Potential Effects of an Exoskeleton-Assisted Overground Walking Program for Individuals With Spinal Cord Injury Who Uses a Wheelchair on Imaging and Serum Markers of Bone Strength: Pre-Post Study
Source: JMIR Rehabil Assist Technol. 2024 Jan 1;11:e53084. doi: 10.2196/53084 (PMC10790203; doi:10.2196/53084)

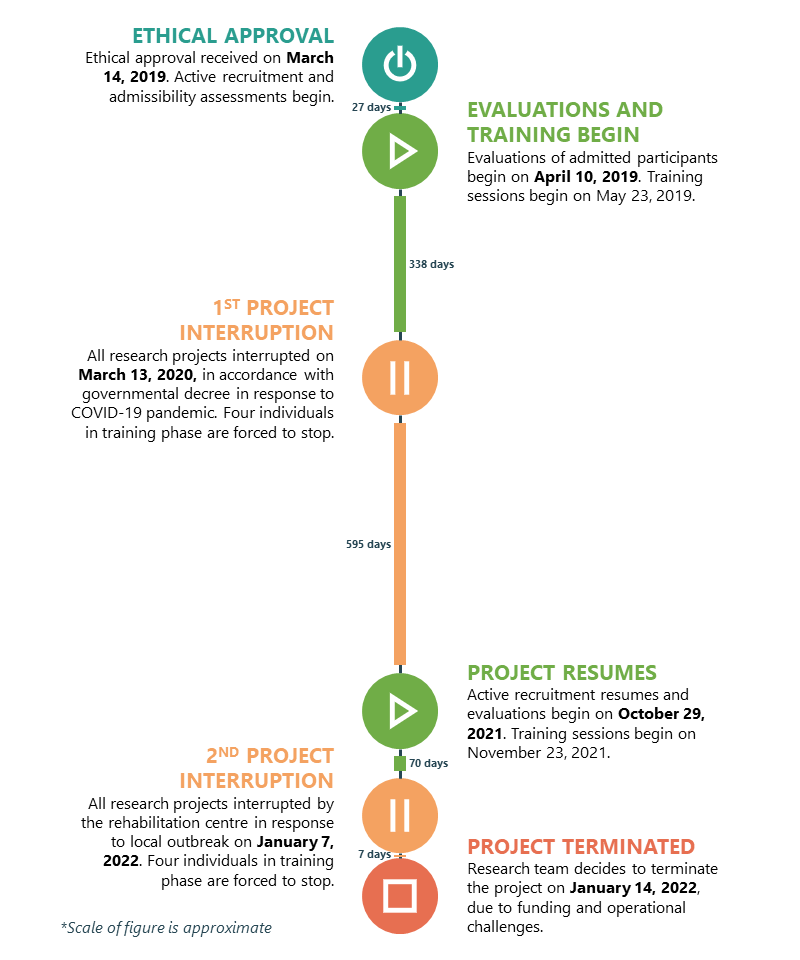

Supplement: Multimedia Appendix 1 [file rehab_v11i1e53084_app1.png]
